# Bacterial microbiota of *Aedes aegypti* mosquito larvae is altered by intoxication with *Bacillus thuringiensis israelensis*

**DOI:** 10.1186/s13071-018-2741-8

**Published:** 2018-03-02

**Authors:** Guillaume Tetreau, Stéphanie Grizard, Chandrashekhar D. Patil, Florence-Hélène Tran, Van Tran Van, Renaud Stalinski, Frédéric Laporte, Patrick Mavingui, Laurence Després, Claire Valiente Moro

**Affiliations:** 1 Université Grenoble Alpes, Laboratoire d’Ecologie Alpine LECA UMR5553, F-38000 Grenoble, France; 2 Centre National de la Recherche Scientifique, Laboratoire d’Ecologie Alpine LECA UMR5553, F-38000 Grenoble, France; 3 Université de Lyon, Lyon, France; 4 Université Lyon 1, Villeurbanne, France; 5 CNRS, UMR 5557, Ecologie Microbienne, Villeurbanne, France; 6 INRA, UMR1418, Villeurbanne, France; 7 School of Life Sciences, North Maharashtra University, Post Box 80, Jalgaon, Maharashtra 425001 India; 8 Université de La Réunion, CNRS 9192, INSERM U1187, IRD 249, Unité Mixte Processus Infectieux en Milieu Insulaire Tropical (PIMIT), Plateforme Technologique CYROI, Sainte-Clotilde, La Réunion, France

**Keywords:** Diptera, *Bacillus thuringiensis*, Denaturing gradient gel electrophoresis (DGGE), Bacterial community fingerprinting, Larval microbiota, Holobiont

## Abstract

**Background:**

Insect microbiota is a dynamic microbial community that can actively participate in defense against pathogens. *Bacillus thuringiensis* (Bt) is a natural entomopathogen widely used as a bioinsecticide for pest control. Although Bt’s mode of action has been extensively studied, whether the presence of microbiota is mandatory for Bt to effectively kill the insect is still under debate. An association between a higher tolerance and a modified microbiota was already evidenced but a critical point remained to be solved: is the modified microbiota a cause or a consequence of a higher tolerance to Bt?

**Methods:**

In this study we focused on the mosquito species *Aedes aegypti*, as no work has been performed on Diptera on this topic to date, and on *B. thuringiensis israelensis* (Bti), which is used worldwide for mosquito control. To avoid using antibiotics to cure bacterial microbiota, mosquito larvae were exposed to an hourly increasing dose of Bti during 25 hours to separate the most susceptible larvae dying quickly from more tolerant individuals, with longer survival.

**Results:**

Denaturing gradient gel electrophoresis (DGGE) fingerprinting revealed that mosquito larval bacterial microbiota was strongly affected by Bti infection after only a few hours of exposure. Bacterial microbiota from the most tolerant larvae showed the lowest diversity but the highest inter-individual differences. The proportion of Bti in the host tissue was reduced in the most tolerant larvae as compared to the most susceptible ones, suggesting an active control of Bti infection by the host.

**Conclusions:**

Here we show that a modified microbiota is associated with a higher tolerance of mosquitoes to Bti, but that it is rather a consequence of Bti infection than the cause of the higher tolerance. This study paves the way to future investigations aiming at unraveling the role of host immunity, inter-species bacterial competition and kinetics of host colonization by Bti that could be at the basis of the phenotype observed in this study.

**Electronic supplementary material:**

The online version of this article (10.1186/s13071-018-2741-8) contains supplementary material, which is available to authorized users.

## Background

*Bacillus thuringiensis* (Bt) are entomopathogenic bacteria widely used as biological insecticides for the control of agricultural pests and disease-carrying insect populations [1]. More than a hundred Bt subspecies have been described to date. Each subspecies produces a specific set of one or more toxins as a crystal during sporulation [2, 3]. After their ingestion by an insect together with bacterial spores, toxins perforate and disrupt the insect gut to allow Bt colonization of the hemocel, ultimately leading to host septicemia [4]. Each Bt subspecies exhibits a high level of specificity toward an insect phylogenetic group [5, 6]. This adaptation to their host with the selection and conservation of key virulence factors makes Bt a pathogen rather than an opportunistic bacterium [7, 8]. Nevertheless, this view has been challenged during the last decade with contrasting reports about the role of the insect gut microbiota on the toxicity and infection capacity of Bt [7]; some authors suggested that host-associated microbiota might be mandatory for Bt to kill insects [9].

The microbiota of insects is a dynamic microbial community that shapes many host life history traits [10–12]. Interestingly, how the microbiota interacts with Bt toxicity and infection process is still far from being elucidated. Previous studies reported possible competition between Bt and microbiota. Bt has been shown to inhibit the growth of gut bacteria by producing bacteriocin [13] while microbiota can also inhibit Bt growth as well as degrade its toxins [14–16]. This protective role of gut microbial community against pathogens colonization, notably by niche occupation, nutrient competition or immune priming, is called “colonization resistance” [11, 17, 18]. Conversely, Bt and microbiota may also exhibit beneficial interactions. For instance, Bt protoxins can be activated into toxins by proteases produced by bacteria from insect gut [19]. Moreover, some opportunistic bacteria, taking advantage of gut damages induced by Bt toxins, can spread across insect tissues and participate to host septicemia [20, 21].

In 2006, Broderick et al. [9] reported that gut microbiota was mandatory for Bt toxicity to gypsy moth larvae. Since then, several studies were conducted and led to contrasting results. Different research groups provided support for a key role of the microbiota on Bt toxicity based on an increased tolerance to Bt after gut bacterial curation by antibiotic treatments [9, 22, 23]. Interestingly, the involvement of gut microbiota seems to be species-dependent [24]. In contrast, some scientists argued that the observed effect is mainly due to the residual antibiotic effect on Bt, resulting in a biased outcome of the experiments [7, 25, 26]. Additional studies performed by other groups found no experimental support for microbiota involvement in Bt toxicity, even on similar insect species [14, 21, 27, 28]. In light of these contrasted results, the relationship between microbiota and Bt toxicity remains unclear and it is therefore a burning topic to address [7]. One might wonder whether these differences are exclusively due to the experimental design (antibiotics used, gut curation procedure) or if they are linked to the gut community itself, known to be highly influenced by the environment and to vary among species [10, 29] and among populations within species [30, 31]. The latter could explain the differences observed from one study to another at both intra- and inter-species levels.

Several studies have already shown that a higher tolerance to Bt is associated with a difference in bacterial community composition [32, 33]. Nevertheless, whether changes in microbiota content are the cause of an increased tolerance or a consequence of Bt infection remains unclear. Moreover, whether microbiota-Bt interaction effect on Bt toxicity is passive or active (i.e. exclusively opportunistic or evolutionary selected) is yet to be investigated. In the present study, we aimed at providing new insights to disentangle these two hypotheses. To do so, we used *Aedes aegypti* mosquito larvae, as the role of dipteran microbiota on Bt toxicity has not been studied to date, and *B. thuringiensis israelensis* (Bti), which is widely used as a biological insecticide to control mosquito populations. To avoid the bias associated with microbiota curation procedures, we studied the dynamics of the microbiota upon exposure to increasing Bt dose, which allowed us to conclude that microbiota modification is a rapid process occurring as a consequence of Bti infection.

## Methods

### Mosquito strain and rearing conditions

Although environment is known to influence microbial community, *Aedes aegypti* larvae are able to shape their microbiota that eventually significantly differ from water’s microbial community [34]. The present work was performed on an *A. aegypti* laboratory strain (Bora-Bora), maintained for years in the laboratory but still exhibiting a high genetic variability [35]. Adults were maintained in standard insectary conditions (27 °C, 14/10 h light/dark photoperiod, 80% relative humidity) in insect rearing cages and fed *ad libitum* with honey as previously described [36]. After reproduction, adult females were blood-fed on mice twice a week and eggs were laid on Whatman paper disposed into crystallizing glass dishes (300 ml capacity) containing tap water. Papers were collected once or twice a week, left to dry and stored at room temperature for less than two months. For the experiment, papers containing eggs from different egg laying dates were put in water containing hay pellets to decrease the amount of oxygen in the water and promote egg hatching. Larvae were reared in tap water and fed with hay pellets in standard insectary conditions.

### *Bacillus thuringiensis israelensis* (Bti) production

To avoid any effect of the insecticide formulation, a non-formulated *Bacillus thuringiensis israelensis* (Bti) toxins/spores suspension was produced in the laboratory using spores isolated from commercial Bti VectoBac WG. Suspensions of spores and crystals were produced on nutrient agar medium as previously described [37]. Full sporulation and crystal production was verified under microscope. The quality of Bti production was assessed on SDS-PAGE. The concentration of the Bti suspensions was determined as the weight of dry pellet divided by the volume of water used for resuspension. The Bti suspension was conserved in water at -20 °C until use.

### Phenotypic characterization of Bti tolerance

Considering that the mosquito strain used is highly susceptible to Bti, using a single dose of Bti would have not allowed discriminating precisely the most tolerant individuals from the most susceptible ones, as they would have all died within a very short time frame. Therefore, mosquito larvae were exposed to an hourly increasing dose of Bti to obtain a phenotypic distribution that maximize the differences between the most tolerant and the most susceptible larvae using the survival time as a proxy [38]. A total of 560 early third-instar larvae were individually isolated in a plastic cup containing 20 ml of tap water. They were disposed in cups the day before the experiment without food to ensure that gut microbial community of all individuals were similar at T = 0. Thirty larvae, referred to as “Control” group, were unexposed to Bti, and were sampled at the beginning of the experiment. The 530 other larvae were exposed to Bti. At T = 0 and every hour afterwards, a dose of 27 μl of a 40 mg/l (1 μg) suspension of Bti spores/crystals was added within each plastic cup. Larval mortality was monitored every 15 min until the sixth hour of experiment and every 30 min onwards. Each dead larva was immediately collected and stored individually in 70% ethanol before performing DNA extraction. The frequent monitoring of larval mortality and the immediate sampling and storage of larvae limited the development of bacteria within larval tissues, which ensures that it did not bias microbiota analyses. The larval instar of each individual was determined during sampling to confirm they all remained as third-instars, and therefore ensured the maintenance of their microbiota. Each larva was labeled with a “T number” corresponding to the time point at which it died, and followed by a second number that gives the replicate number whenever several larvae died at the same time point (e.g. T4-5 is the fifth larva that died after four hours of exposure to Bti). Based on the phenotypic distribution, larvae exposed to Bti were grouped in three different categories based on their tolerance level: the 20 larvae that died during the first 6 h were qualified as “Susceptible”, the 30 larvae that survived more than 11 h were qualified as “Tolerant”, and the remaining ones (480 larvae in total) that survived between 6 and 11 h as “Intermediate”.

### DNA extraction from larvae

A total of 15, 18, 15 and 20 larvae from the “Control”, “Susceptible”, “Intermediate” and “Tolerant” groups, respectively, were analyzed. Prior to DNA extraction, larvae were surface-disinfected to avoid any environmental contamination. Individuals were rinsed three times in sterile water, surface-disinfected for 5 min with 70% ethanol, and rinsed five times with sterile water. DNA extraction from larvae was slightly adapted from Minard et al. [39] who worked on adult mosquitoes. Considering that most, if not all, bacterial microbiota is contributed by the gut [30], gut larvae were not dissected. DNA extraction was performed on whole larvae to avoid potential bias induced by the dissection process (e.g. partial loss of gut content during extraction). Briefly, each larva was introduced into a 2 ml Eppendorf tube containing 5 mm diameter inox beads, plunged into liquid nitrogen for 10 s, and then crushed twice for 45 s using a Bioblock scientific MM 2000 mill (Retsch, Eragny sur Oise, France). After incubation with the extraction buffer, each sample was treated with 4 μl of RNase (100 mg/ml), and then kept at 37 °C for 5 min. Lipids and proteins were extracted with phenol-chloroform-isoamyl alcohol (25:24:1; v/v/v) and chloroform-isoamyl alcohol (24:1; v/v) steps, and DNA extracted with isopropyl alcohol. Samples were then centrifuged for 50 min at 13,200× *rpm* at 4 °C. DNA pellets were rinsed twice with 75% cold ethanol, air-dried under laminar flow, and re-suspended in 20 μl of TE buffer (10 mM Tris, 1 mM EDTA). The quantity of DNA was measured based on the absorbance at 260 nm and its quality was assessed by the absorbance ratio A260/280 and A260/230 (SAFAS UVmc2, Monaco).

### PCR amplification of *16S* rRNA fragments

A nested PCR approach was used to generate the PCR-DGGE profiles from previously extracted larval genomic DNA. The first PCR reaction was performed using the primer set pA (5'-AGA GTT TGA TCC TGG CTC AG-3') and pH (5'-AAG GAG GTG ATC CAG CCG CA-3') [40]. PCR amplifications were carried out in 25 μl. Each reaction contained 23 μl of PCR mix which included: 200 μM of dNTPs, 0.5 μM of each primer (Thermo Fisher Scientific, Illkirch, France), 0.025 mg/ml of T4 gene 32 Protein (Roche, Boulogne-Billancourt, France), and 0.126 U/μl of Expand High Fidelity Enzyme Mix in 1× Expand High Fidelity Buffer containing MgCl2 (Roche), and nuclease-free water. Reactions were completed with 2 μl of DNA template (30 ng/μl). The amplification was carried out by performing an initial denaturation step at 95 °C for 3 min, followed by 35 cycles at 94 °C for 30 s, 55 °C for 40 s, and 72 °C for 90 s, and finalized by an extension step at 72 °C for 10 min.

The second PCR reaction targeted a fragment of approximately 200 bp of the bacterial conserved V3 region of *16S* rRNA genes and was performed with a broad range bacterial primer set V3F-GC (5'-GCC GCC CGC CGC GCG CGG CGG GCG GGG CGG GGG CAC GGG GGG ACT CCT ACG GGA GGC AGC AG-3') and V3R (5'-ATT ACC GCG GCT GCT GG-3') [41, 42]. The nested PCR was carried out in 50 μl volume containing 3 μl of the first PCR run as template. Each PCR reaction contained 200 μM of dNTPs, 0.5 μM of each primer, 0.025 mg/ml of T4 gene 32 Protein, 0.04 U/μl of TaqDNA polymerase (Invitrogen) in 1× reaction PCR buffer without MgCl_2_, 1.5 mM MgCl_2_, and nuclease-free water. Amplification started by a denaturation step at 94 °C for 2 min, first followed by seven cycles at 94 °C for 30 s, 55 °C for 30 s, and 72 °C for 1 min, second by 21 cycles at 92 °C for 30 s, 55 °C for 30 s, and 72 °C for 80 s, and finalized by an extension step at 72 °C for 10 min. All PCRs were run in a T Gradient thermocycler (Biometra, Göttingen, Germany). PCR product concentrations were assessed as previously described. Negative and positive controls were added to each PCR mix.

### DGGE community fingerprinting

DGGE procedure was conducted using the Ingeny PhorU system (Apollo Intruments, Compiègne, France) as previously described [43]. PCR products obtained from the V3 region amplification (3.5 μg per lane) were loaded onto 6% (w/v) polyacrylamide gel containing a 35–65% denaturant gradient of urea and formamide, and run in 1× TAE buffer at 60 °C for 16 h at 100 V. All gels were run with the same reference marker (1 kb Plus Ladder, Thermo Fisher Scientific) for normalization purpose in computer analyses. After the run, gels were stained using SYBR green (Thermo Fisher Scientific) for 30 min in the dark, rinsed with water, and photographed under UV-light. DGGE profiles were digitized using a camera and stored as TIFF files for computer analyses.

### Sequencing of DGGE fragments and sequence analyses

The most abundant DGGE bands and some bands showing differences between conditions were excised from the gels with a sterile scalpel and rinsed individually three times in sterile ultra-pure water. Bands were incubated at 65 °C in the last washing solution and allowed to diffuse overnight at 4 °C in 100 μl of sterile water. Two microliters of eluate from individual bands were used to reamplify PCR products using the same bacterial primer set, except that primer V3F did not contain the GC clamp. Reaction conditions were the same as those described above. PCR products were sequenced at Biofidal-DTAMB (FR Bio-Environment and Health, Lyon, France). The sequences were analyzed with the BLASTN program. Sequences obtained were assigned to KC867313, KY124158, KM488465, KY608158, JQ58869, KY608117, FR821125, KY608117, KY124158 accession numbers in the GenBank database.

### Analysis of DGGE patterns and statistical analyses

Gels were normalized using BioNumerics v.7.1 software (Applied Maths, Sint-Martens-Latem, Belgium). After normalization, a unique matrix containing the information related to the presence/absence of each band as well as its relative intensity was generated for all samples. Community composition and structure of each treatment group were compared by clustering lanes by the Jaccard similarity coefficient implemented in the software and using the unweighted-pair group method with arithmetic mean (UPGMA), rolling-disk background subtraction, and optimization at 0.5% [44–46]. Dice similarity coefficient was also tested and gave similar outputs (data not shown). This band-matching surface matrix was used as support to further DGGE profile analyses.

To investigate a potential negative correlation between intensity of bands corresponding to Bti and to *Acinetobacter*, Principal Components Analysis (PCA) was performed using *princomp* function in R 2.14.1 software [47]. The band-matching surface matrix of the two bands of Bti (at 312 and 288 bp) and the two bands of *Acinetobacter* sp. (at 681 and 649 bp) were used as input and correlation circles were generated. For each band, the cosinus was calculated as a proxy of the linear correlation with the first three components. Cosinus^2^ were also calculated to indicate the quality of their representation by each component (from 0, bad representation; to 1, excellent representation).

The band-matching surface matrix including all bands was used to calculate Shannon and Simpson’s diversity indices and Pielou’s evenness values, and to analyze community structure by exporting it into PRIMER-E v.6.1.16 software (PRIMER-E Ltd, Plymouth, UK). The resemblance matrix was obtained using Bray-Curtis dissimilarity and nonmetric multidimensional scaling (NMDS) graphs were generated from our dataset previously modified by a fourth-root transformation [48]. The values from Bray-Curtis index varies from 0 (similar) to 1 (completely dissimilar community composition). Euclidian distances were also tested to build the resemblance matrix and gave similar results (data not shown). An associated statistical analysis was performed based upon the analysis of similarity (ANOSIM, one-way analysis, 5000 permutations). The associated global R described the percentage of permutations related to the *P*-value, and the stress value indicated how faithful the relationships among samples are represented in the ordination plot.

In parallel to community structure analyses, the number of bands (taken as indicative for species richness), the Shannon (H’) and Simpson (1-λ') indices of diversity (with Simpson’s index being given more weight on dominant species compared to Shannon’s), and the Pielou’s index (a measurement of the community evenness) were used, which all together offer an insightful picture of the community α-diversity [49, 50]. Indices were all checked for normality and transformed when necessary. Only Simpson index values required log-transformation. Average differences between treatment groups were analyzed with linear models (package stats, [47]). Whenever ‘groups’ explained significant variation, *post-hoc* Tukey’s tests were used to compare pairs of groups (package multcomp, [51]).The normality of residuals was tested using Shapiro tests. Mean values of models and other averages were reported with their standard error. Statistical tests were based on threshold α = 0.05 and considered significant when *P* < 0.05. Analyses were conducted in R v.2.14.1 software [47].

## Results and discussion

**Fig. 1:**
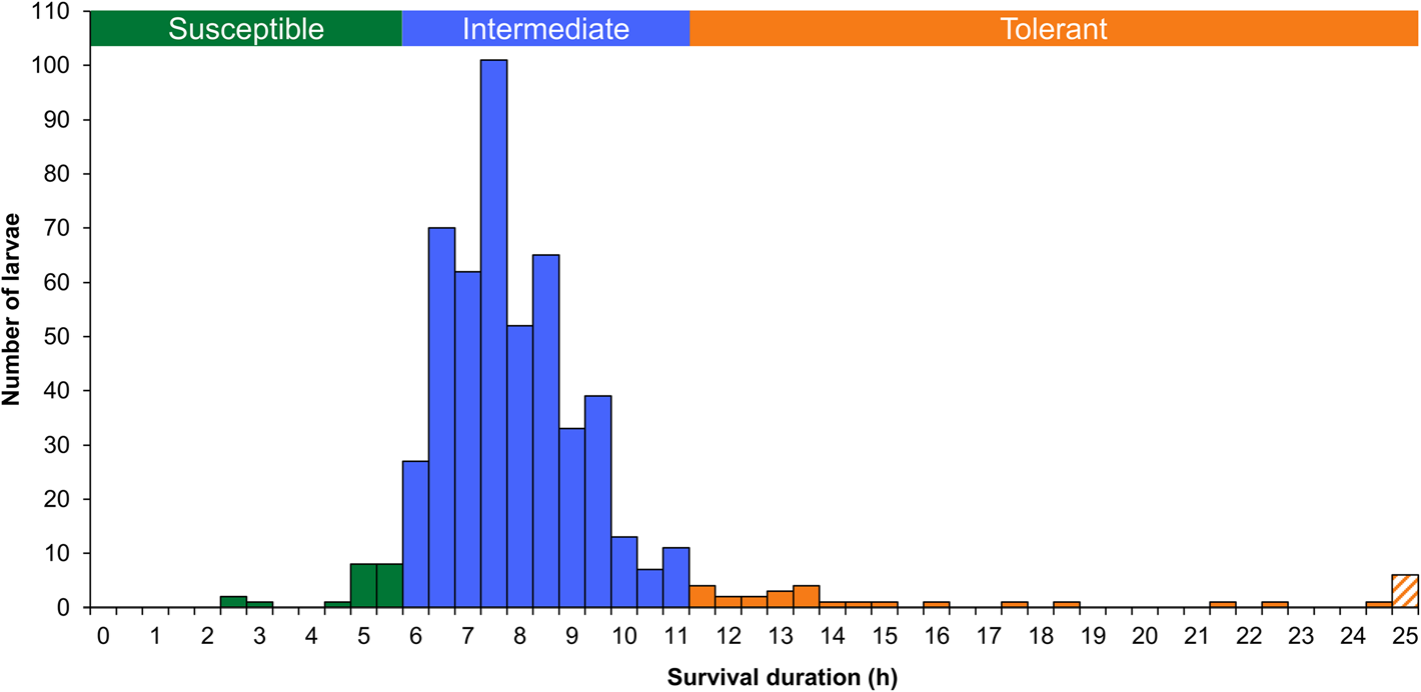
Phenotyping of mosquito larvae exposed to Bti. A dose of 1 μg suspension of Bti spores/crystals was applied every hour to each of the 530 larvae individually disposed in plastic cups containing 20 ml of tap water. Thirty larvae were sampled before exposure to Bti and formed the “control” group. The number of dead larvae sampled at each time point is indicated in the figure. Larvae were separated into three subgroups: “Susceptible” (larvae dead in less than 6 h of exposure to Bti), “Intermediate” (larvae dead between 6 and 11 h) and “Tolerant” (larvae dead after 11 h), which are represented in green, blue and orange, respectively. The bar hatched corresponds to six larvae still alive after 25 h of exposure to Bti

Larvae from the Bora-Bora *Ae. aegypti* mosquito strain were individually exposed to an hourly increasing dose of Bti (1 μg suspension of Bti spores/crystals applied every hour) in order to separate the most susceptible individuals from the most tolerant ones (Fig. 1). While this mosquito strain has been reported as highly susceptible to Bti [52], our phenotyping experiment revealed that individual survival upon Bti exposure was highly variable, with some larvae dying after 2.5 h of exposure while others survived 25 h exposure to a final dose of Bti 15 times greater. Larvae were attributed to three different groups based on their level of susceptibility (Fig. 1): the twenty most susceptible and the thirty most tolerant were attributed to “Susceptible” and “Tolerant” groups, respectively, while the 480 others (90.6% of the total number of exposed larvae) belonged to the “Intermediate” group. A fourth “Control” group was composed of unexposed larvae sampled at T = 0.

**Fig. 2:**
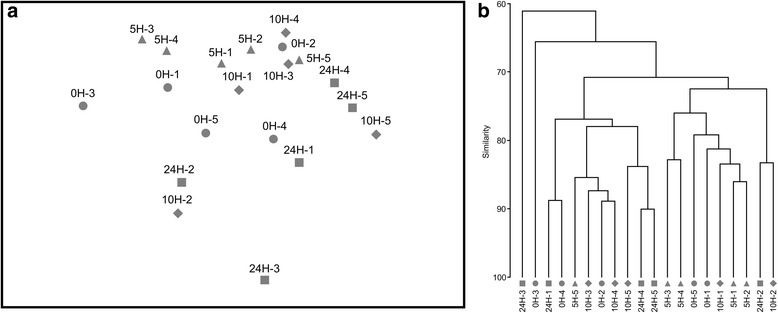
Analysis of the evolution of bacterial communities of unexposed mosquito larvae over time. **a** Non-metric multi-dimensional scaling plot (NMDS) of larval bacterial communities based on DGGE profile analysis (2D stress = 0.12). **b** Hierarchical clustering (group average method) of the samples. NMDS and clustering were based on fourth-root transformed distances obtained using Bray-Curtis dissimilarity index. Distances were calculated based on DGGE gel provided in Additional file 1: Figure S1. Data from unexposed individuals sampled at 0, 5, 10 and 24 h are represented by circles, triangles, diamonds and squares, respectively. Sample names are constituted of the time of sampling associated with the replicate number

The stability of the microbiota of third instar larvae throughout the 24 h time frame of the experiment was verified by maintaining unexposed larvae during 0, 5, 10 and 24 h in the same laboratory conditions as the phenotyping experiment. As expected, bacterial communities did not change over time in unexposed larvae (Fig. 2, Additional file 1: Figure S1). It is well known that microbiota drastically change between developmental stages, and even between instars in the same stage [11, 53–55]. Nevertheless, it is quite stable within the same instar as long as the environment, which is known to be one of the major factor of microbiota shaping, is stable [11, 56]. The laboratory offers a fully controlled environment and the larvae were maintained overnight prior the experiment for ensuring that their bacterial community was stabilized. Therefore, the “Control” group is representative of the mosquito laboratory larval microbial community in the absence of insecticide and is stable throughout the 24 h time frame of the experiment.

**Fig. 3:**
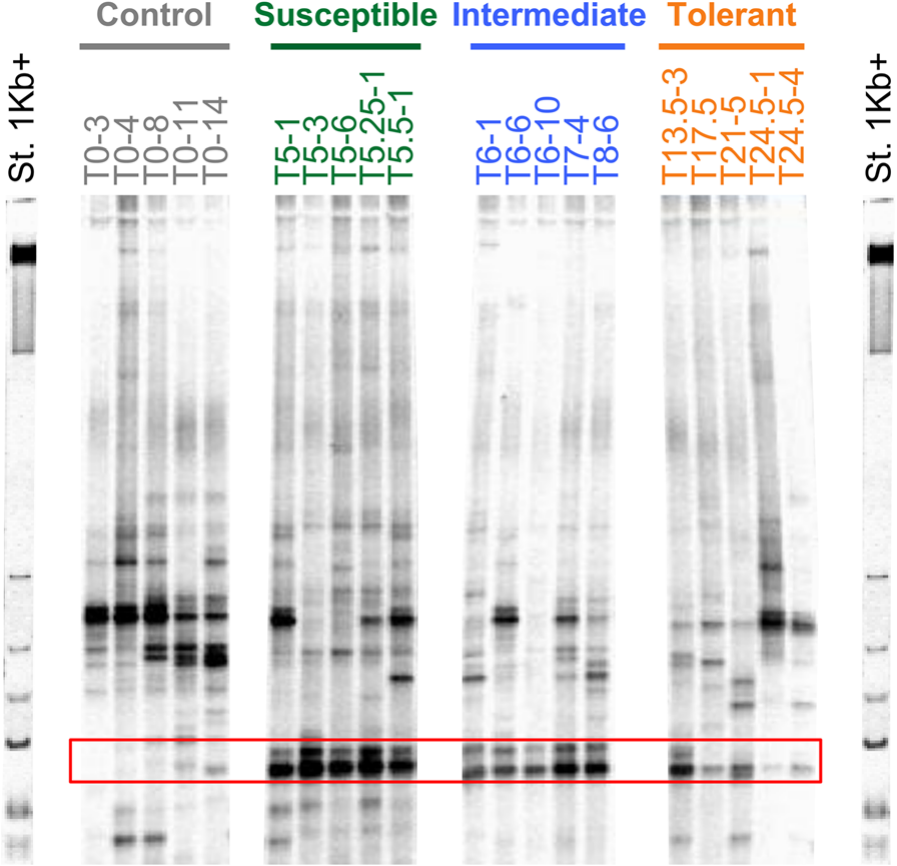
DGGE profiles of bacterial communities of *Ae. aegypti* larvae depending on their Bti tolerance level. Five representative individuals are shown per category. Each larva was labeled with a “T number” corresponding to the time point at which it died (see Fig. 1). It was followed by a second number that gives the replicate number whenever several larvae died at the same time point. Four gels were performed to analyze a total of 15, 18, 15, and 20 individual larvae from the “Control”, “Susceptible”, “Intermediate” and “Tolerant” groups, respectively (Additional file 5: Table S3). St. 1 kb + ladder was used as an external gel migration control. Several bands were excised and sequenced to identify to which bacterial genus they were affiliated (Additional file 1: Figure S1). The two bands corresponding to Bti are indicated by a red rectangle (Additional file 1: Figure S1)

To investigate the composition and diversity of bacterial communities of larvae from each group, PCR-DGGE fingerprints of the hypervariable V3 region of the *rrs* gene were produced from extracted genomic DNA larvae (Fig. 3). Some representative DGGE bands were excised from the gel, re-amplified and sequenced. Among the single sequences, BLAST analyses revealed that sequences were affiliated mostly with *Bacillus*, *Acinetobacter*, *Staphylococcus* and *Delftia* (Additional file 2: Figure S2).

Two bands were identified as Bti in the DGGE profiles (Fig. 3, Additional file 2: Figure S2). These bands were absent from profiles of most unexposed larvae, which is expected for the “Control” group (Fig. 3, Table 1). These bands were found in 89% and 100% of DGGE profiles of individuals from “Susceptible” and “Intermediate” groups, respectively. Their relative intensity ranged from 8.2 to 12.4% of total bands intensity of the profiles (Table 1). Interestingly, Bti bands were found in only 65% of “Tolerant” profiles. While the relative intensity of the higher band of Bti in “Tolerant” was 2.3 and 1.5-fold lower than the same one in “Susceptible” and “Intermediate” groups, respectively, the lower band had a similar relative intensity in all three exposed groups (11.8–12.7%, Table 1). Therefore, an overall decrease of the number of individuals with detectable Bti and of the proportion of Bti in the detectable bacterial community was observed in the most tolerant individuals compared to the most susceptible ones (Table 1). Considering that they were exposed to hourly increasing doses of Bti, the most tolerant larvae were exposed to higher doses for a longer time. If the larvae that survived longer (“Tolerant” group) did not immunologically respond to impede Bti infection, one could expect an increase in the proportion of Bti in the bacterial community, and we observed the opposite here. This suggests that, in spite of fresh Bti spores and crystals suspension added every hour to the water, the most tolerant larvae were able to control the infection, leading to a partial to complete clearance of Bti from their body. Nevertheless, at this point, these results do not allow determining whether Bti clearance is driven, at least partially, by the bacterial microbiota or exclusively under control of the larval immune system.

**Table 1:** Analysis of mean relative abundance of bands corresponding to Bti from DGGE profiles. The presence of band shows the number of individuals with the band on their profile over the total number of individuals analyzed (percentage in parenthesis). Relative intensity is the mean (± SD) of the intensities of Bti band divided by the total intensity of all bands analyzed from each individual. Higher band and lower band of Bti correspond to 312 bp and 288 bp fragments, respectively, that were both identified as Bti by sequencing (Additional file 1: Figure S1)

Treatment	Higher band of Bti		Lower band of Bti	
	Presence of band (%)	Relative intensity (%)	Presence of band (%)	Relative intensity (%)
Control	1/15 (7)	0.46 ± 1.71	1/15 (7)	0.12 ± 0.43
Susceptible	16/18 (89)	13.08 ± 6.40	16/18 (89)	12.06 ± 8.87
Intermediate	15/15 (100)	11.80 ± 3.26	15/15 (100)	11.80 ± 3.26
Tolerant	13/20 (65)	5.71 ± 6.65	13/20 (65)	12.70 ± 14.38

**Fig. 4:**
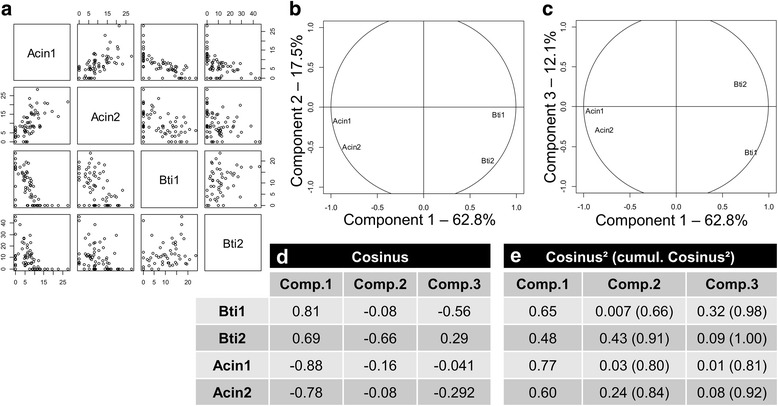
Principal Components Analysis (PCA) of bands corresponding to Bti and to *Acinetobacter* sp. (Additional file 1: Figure S1). The two bands for Bti were noted Bti1 (312 bp) and Bti2 (288 bp) and those for *Acinetobacter* sp. were Acin1 (681 bp) and Acin2 (649 bp). **a** 2D distribution of all samples analyzed in function of all pairs of bands analyzed (Bti1, Bti2, Acin1, Acin2). **b**, **c** PCA correlation circles in function of the first two components (**b** cumulated explained variance of 80.3%) and of the first and third components (**c** cumulated explained variance of 74.9%) showing a negative correlation between the two Bti and the two *Acinetobacter* band intensities. **d** Linear correlation of each band with the first three components, expressed by their cosinus, indicated a high level of positive (Bti1 and Bti2) and negative (Acin1 and Acin2) correlation with the first component. **e** Quality of representation of the four bands is indicated by their cosinus^2^ (varies from 0 to 1, 1 being the best representation). This indicates that the four bands are well explained by the first three components

Interestingly, bands corresponding to *Acinetobacter* were negatively correlated to those of Bti (Fig. 4). While *Acinetobacter* was frequently associated with the “Control” group at a high intensity, the bands tended to decrease in intensity or disappear in the three other modalities raising questions about the possible antagonistic interaction between this bacterium/bacterial genus and Bti. *Acinetobacter* are ubiquitous bacteria that can be found in water, soil and living organisms [57]. So far, only one study has reported the role played by *Acinetobacter* in the biology of a fly insect species *Stomoxys calcitrans*, for which these bacteria were required to ensure the complete development of larvae [58]. Nonetheless, and in accordance with a potential antagonistic role, the *Acinetobacter* sp. strain KNF2022 was previously shown to produce an antiviral compound with inhibitory effects on the tobacco mosaic virus [59]. Members of *Acinetobacter* are hosted by a number of mosquito species [39]. However, most studies on mosquito-associated bacterial communities focused on adult stage and solely few studies have attempted to assess the infection status of *Acinetobacter* in larval stages. Recently, David et al. [60] showed that *Acinetobacter* species belonged to the core bacterial microbiota associated with *Ae. aegypti* as the mosquito stably harbors this bacterium throughout its lifespan.

**Fig. 5:**
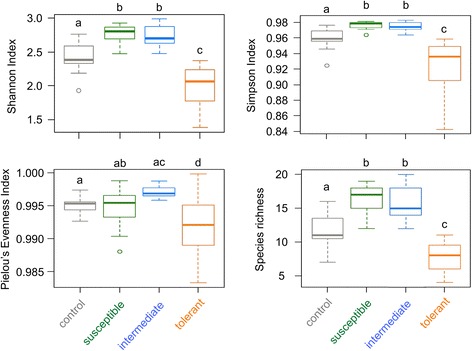
Boxplot representation of Shannon and Simpson’s diversity, and Pielou’s evenness indices and of species richness. Species richness was represented by the number of bands. Significant differences between the four groups (“Control”, “Susceptible”, “Intermediate” and “Tolerant”) were investigated by ANOVA followed by multiple pairwise comparisons of means (*post-hoc* Tukey’s HSD test) using R software version 2.14.1 [47]. Different letters indicate significant differences between groups. Values from all statistical tests are available in Additional file 3: Table S1

Four diversity indices were calculated based on DGGE profiles to provide an overview of the larval bacterial community within each group (Fig. 5): species richness (total number of bands), species diversity (Shannon and Simpson indices), and species evenness (Pielou’s index). All groups significantly differed from each other, except those bearing “Susceptible” and “Intermediate” larvae (Fig. 5, Additional file 3: Table S1). The most tolerant larvae had the lowest species richness and diversity indices, and the lowest evenness index, indicating that few bacterial species became dominants (Additional file 4: Table S2). In addition, the resemblance matrix upon DGGE lanes showed that bacterial communities associated with tolerant larvae had the lowest similarity among each other (Additional file 5: Table S3). This indicates that the bacterial communities from tolerant larvae strongly differed from one individual to another.

**Fig. 6:**
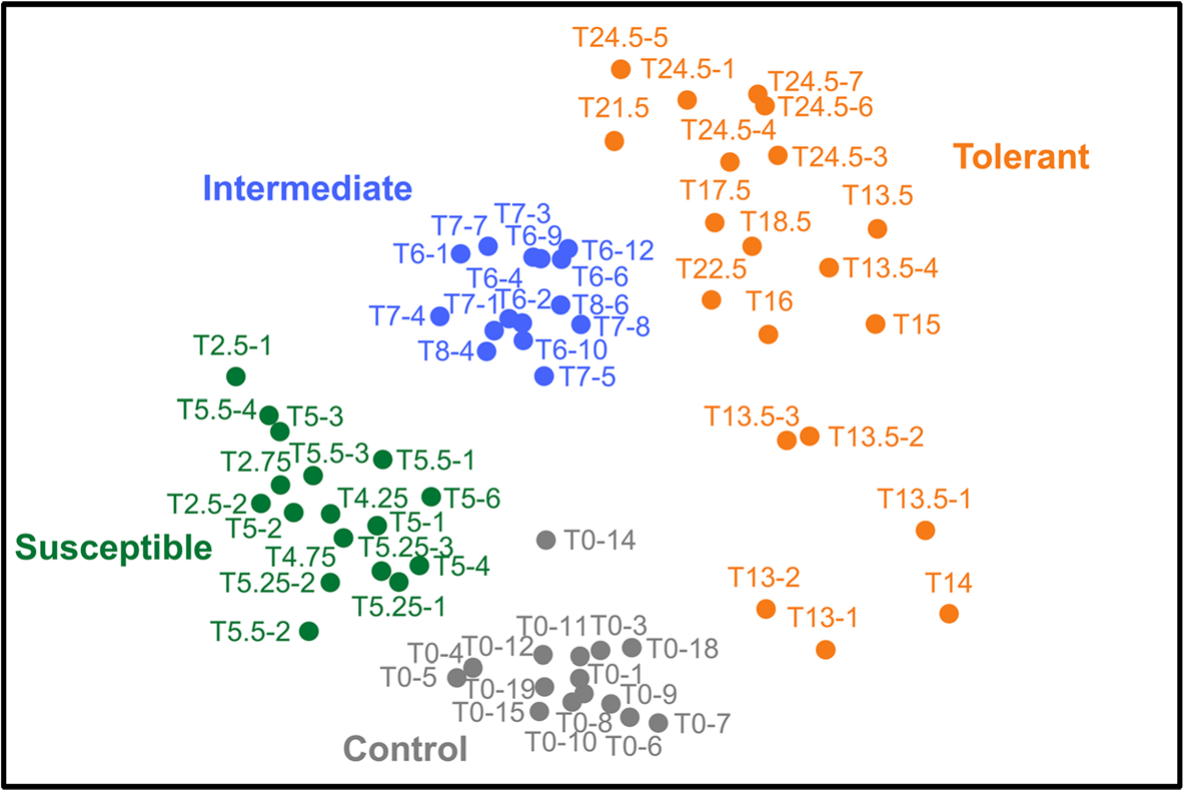
Non-metric multi-dimensional scaling plot (NMDS) of larval bacterial communities based on DGGE profile analysis. The 2D stress of the NMDS was 0.21. Graphs were generated from fourth-root transformed distances obtained using Bray-Curtis dissimilarity index. Data from the “Control”, “Susceptible”, “Intermediate” and “Tolerant” groups are represented in grey, green, blue and orange circles, respectively. Sample names and groups as in Fig. 2. Three-dimensional scaling plots representations of NMDS (3D stress = 0.14) are available in Additional file 6: Figure S3

Based on the resemblance matrix generated from DGGE profiles, pairwise comparisons from the ANOSIM analysis confirmed that all groups significantly differed from each other (Table 2). The clustering patterns observed in the non-metric multi-dimensional scaling (NMDS) plots revealed that bacterial community structure from each group did not overlap with each other (Fig. 6, Additional file 6: Figure S3). Strikingly, after only few hours of Bti exposure, the bacterial communities of the most susceptible larvae already harbored a bacterial microbiota significantly different from the control ones (Table 1, Fig. 6). The NMDS representation confirmed the higher dispersion among the “Tolerant” group compared to other groups (Fig. 6, Additional file 4: Table S2, Additional file 5: Table S3). Furthermore, this higher dispersion across microbiota of the most tolerant individual larvae seems to be associated with the survival time as there is a gradient from the larvae that survived 13–14 hours to the ones that survived 24.5 hours in the NMDS representation (Fig. 6). This cannot be dissociated from a potential dose-effect, considering that Bti concentration increased over time. Last but not least, the “Control” group is not embracing all the other groups but is rather separated with low dispersion in the NMDS (Fig. 6, Additional file 7: Figure S4). This observation excludes the selection in the more tolerant individuals of a specific bacterial community from the diversified microbiota present before exposure, otherwise “Control” individuals would have been spread in the NMDS analysis and be overlapping the other conditions. The pattern observed suggests that after exposure to Bti, the bacterial community is deeply affected and modified, comparatively to unexposed larvae. Therefore, bacterial microbiota composition associated with different levels of larval tolerance is here not the cause of the level of tolerance but rather a consequence of Bti infection.

**Table 2:** R-values and significance level of pairwise comparisons from one-way analysis of similarities (ANOSIM) of DGGE profiles

	Control	Susceptible	Intermediate
Susceptible	0.770***		
Intermediate	0.984***	0.897***	
Tolerant	0.680***	0.782***	0.489***

***P < 0.001

*Notes*: Global R statistic = 0.722; Number of permutations = 5000. R ranges from 0 (no differences) to 1 (all dissimilarities between larval bacterial communities of the different treatment groups are larger than any dissimilarity within their own group)

It is noteworthy that control larvae were sampled alive while most exposed larvae were sampled dead, as a consequence of the phenotyping procedure. One could argue that the microbiota of the larvae begins to change when larvae approach to death and that this could explain the differences observed between the different larvae. This effect seems to be negligible considering that the most tolerant larvae still alive after 25 hours of exposure exhibited bacterial communities highly comparable to those dying only few hours before.

Based on our results, a scenario of the impact of the Bti infection on bacterial microbiota can be depicted. After the first exposure, microbiota began to change upon Bti ingestion while the most susceptible larvae died (Fig. 2). Only the most tolerant larvae survived by getting rid of, or significantly reducing, the quantity of Bti present in their body (Fig. 3). This process is likely to be unrelated to the microbiota, considering that no key bacteria taxa were found to be over-abundant in the “Tolerant” group in comparison with the “Control” one. In a recent similar experiment, exposure of larvae from the same laboratory Bora-Bora strain of *A. aegypti* to sublethal doses of Bti spores and crystals induced a significant 2.5- and 4.9-fold increased gene expression of two antimicrobial peptides (AMPs), a defensin and a cecropin, respectively (Table S3 in [61]). This suggests that larvae might directly control Bti by producing antimicrobial peptides (AMPs). While cecropin are generally more active against GRAM negative than GRAM positive bacteria such as Bt [62], anti-Bt activity of defensins has already been evidenced in lepidopterans [63] and coleopterans [64]. Such an increased AMP gene expression has also been characterized in other insects exposed to different Bt subspecies [65–68]. In addition, the involvement of AMPs in tolerance to Bt has been functionally validated by RNAi experiments [69]. Consistent with our results, this could indicate that insect larvae exposed to both toxins and spores might trigger an increased immune response to clear off bacteria out from host body while healing their gut damaged by the toxins [70]. Increased AMPs expression can be at the basis of the strong decreased diversity and evenness of the bacterial communities observed in the “Tolerant” larvae (Fig. 5). The impact of the immune response of the most tolerant larvae on their microbiota might be time-dependent and/or dose-dependent considering that larvae from “Tolerant” group were collected during a wide range of time (from 11 to 25 h), therefore exposed to different amounts of Bti spores/crystals. Altogether, our results suggest that a modified microbiota is associated with a higher tolerance of mosquitoes to Bti but that it does not seem to be responsible for it. Higher tolerance is rather due to insect intrinsic, probably genetic, mechanisms yet to be further characterized [61].

Experiments linking kinetics of expression of candidate genes for immunity and overall microbial communities (e.g. by including fungi together with bacteria) are needed to better understand the association between microbiota diversity and mosquito tolerance. Although apparently not playing a direct role, bacteria may act afterwards during spore germination in larvae. Moreover, considering that immune priming induced by Bt exposure is transmissible to the next generation [71, 72] and that AMPs are driving gut colonization by symbiotic bacteria during the embryonic development [73], performing the same experiments on successive generations might allow identifying long term adaptation to Bt and uncover a potentially unexpected role of gut microbiota in such trans-generational Bt adaptation.

## Conclusions

The role of microbiota in the toxicity of Bt to insects is still a burning question to be solved. It has been investigated in several species, mostly lepidopterans, by using antibiotics to cure insect gut from bacteria. Nevertheless, this approach is controversial, due to the direct effects of antibiotics on the insect physiology. Here, we provided an alternative approach to study this question, by exposing the larvae to increasing doses of Bti and comparing the microbiota of the most susceptible from that of the most tolerant individuals. Moreover, this allowed us to investigate whether a modified microbiota is the cause or a consequence of Bt exposure, which remained an open question. Our study was conducted on mosquito larvae with the *israelensis* subspecies of Bt, which has never been used previously on this topic. Our results revealed that a modified microbiota is associated with a higher tolerance of mosquitoes to Bti. Bacterial microbiota from the most tolerant larvae showed the lowest diversity but the highest inter-individual differences. The proportion of Bti in the host tissue was reduced in the most tolerant larvae as compared to the most susceptible ones, suggesting an active control of Bti infection by the host. Such modification of bacterial microbiota seems to be rather a consequence of Bti infection than the cause of the higher tolerance. This study paves the way to future investigations aiming at unraveling the role of host immunity, inter-species bacterial competition and kinetics of host colonization by Bti that could be at the basis of the phenotype observed in this study.

## Additional files

Additional file 1: **Figure S1.** Picture of the DGGE gel containing unexposed larvae sampled at 0, 5, 10 and 24 h. (PDF 108 kb)

Additional file 2: **Figure S2.** Composite picture of a DGGE gel containing all four groups of larvae. Bands excised and sequenced are indicated by a red rectangle. The corresponding species identified are indicated in the table. (PDF 242 kb)

Additional file 3: **Table S1.** Tukey’s *post-hoc* test outputs from larval bacterial microbiota. Linear models were used to test the relationship between alpha-diversity metrics (diversity) and treatment groups (treatment). Tukey’s *post-hoc* tests were used to compare pair of treatment (*Z*-tests). *P*-values are then reported for each model and indicated in bold when significant (*P* < 0.05). (PDF 195 kb)

Additional file 4: **Table S2.** Band counting numbers, Shannon index (diversity), Simpson index (diversity) and Pielou’s index (evenness). They are based on DGGE band-matching surfaces. Results are for larvae never exposed to *Bacillus thuringiensis israelensis* (Bti) (“Control”) and for larvae exposed to Bti, including larvae dead in less than 6 h of exposure to Bti (“Susceptible”), in between 6 and 11 h (“Intermediate”), and past 11 h (“Tolerant”). Results are given with mean (± standard deviation) (minimum-maximum values). (PDF 277 kb)

Additional file 5: **Table S3.** Bacterial community similarity within and between treatment groups. Similarity values are based on DGGE band-matching surface matrix. Results are presented as the mean (± standard deviation) (minimum-maximum values). (PDF 9 kb)

Additional file 6: **Figure S3.** Pictures of the four DGGE gels used in the analysis. (PDF 142 kb)

Additional file 7: **Figure S4.** Non-metric multi-dimensional scaling plots of the bacterial communities of larvae based on DGGE lane analysis. 3D stress of the NMDS was 0.14. The NMDS representation is based on a Bray-Curtis dissimilarity matrix. Correspondence of sample names is indicated in the legend of Fig. 2. (PDF 65 kb)

## Abbreviations

AMP: antimicrobial peptide
ANOSIM: analysis of similarity
Bt: *Bacillus thuringiensis*
Bti: *Bacillus thuringiensis israelensis*
DGGE: denaturing gradient gel electrophoresis
NMDS: non-metric multi-dimensional scaling
PCA: principal components analysis
RNAi: interference RNA
