# Supplementary figures and images for: Bacterial microbiota of Aedes aegypti mosquito larvae is altered by intoxication with Bacillus thuringiensis israelensis
Source: Parasit Vectors. 2018 Mar 2;11:121. doi: 10.1186/s13071-018-2741-8 (PMC5834902; doi:10.1186/s13071-018-2741-8)

**Additional file 1: Figure S1.** Picture of the DGGE gel containing unexposed larvae sampled at 0, 5, 10 and 24 h.

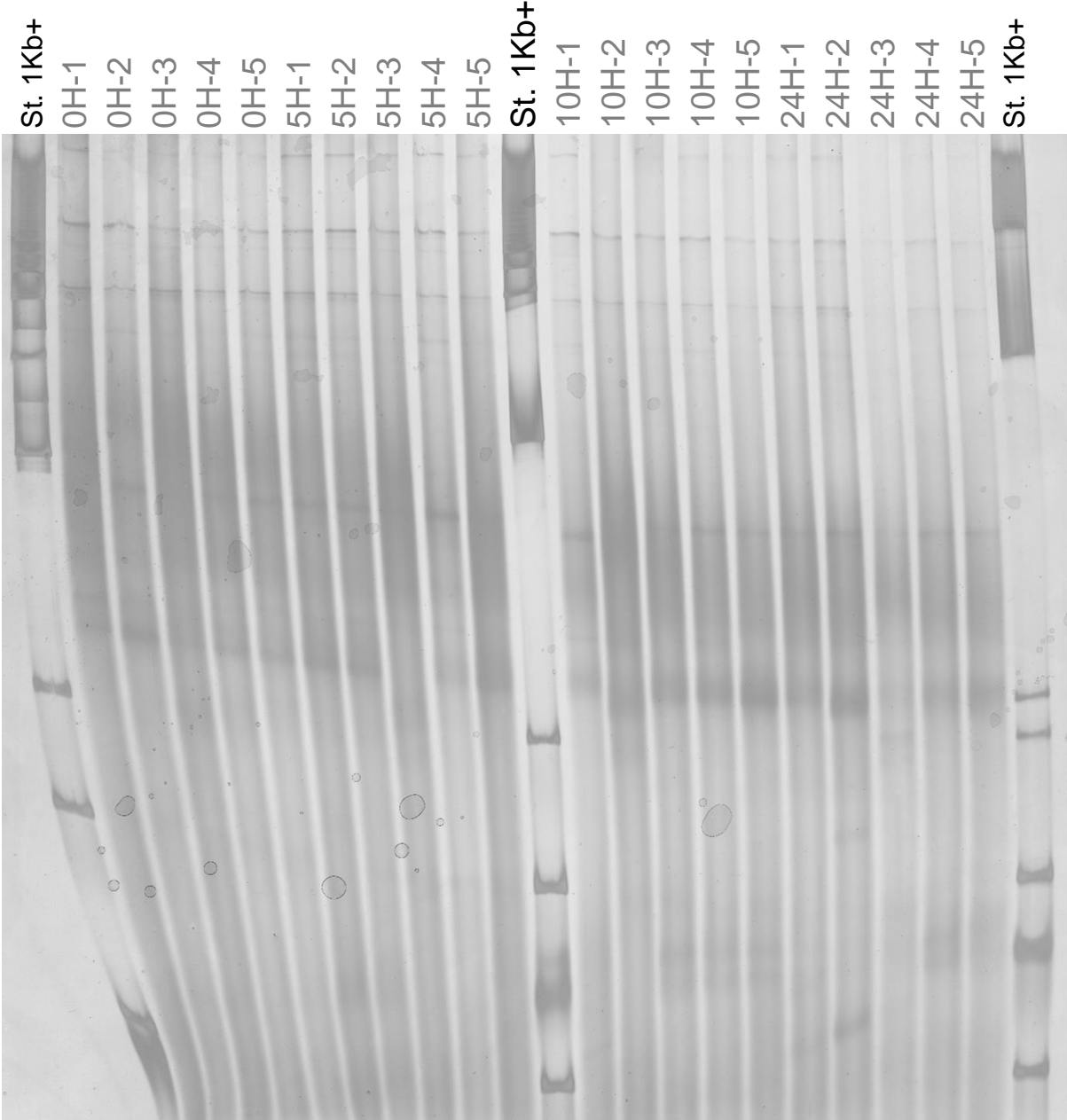

Supplement: Supplementary file 1 — Figure S1. Picture of the DGGE gel containing unexposed larvae sampled at 0, 5, 10 and 24 h. (PDF 108 kb) [file 13071_2018_2741_MOESM1_ESM.pdf]

**Additional file 6: Figure S3.** Pictures of the four DGGE gels used in the analysis.

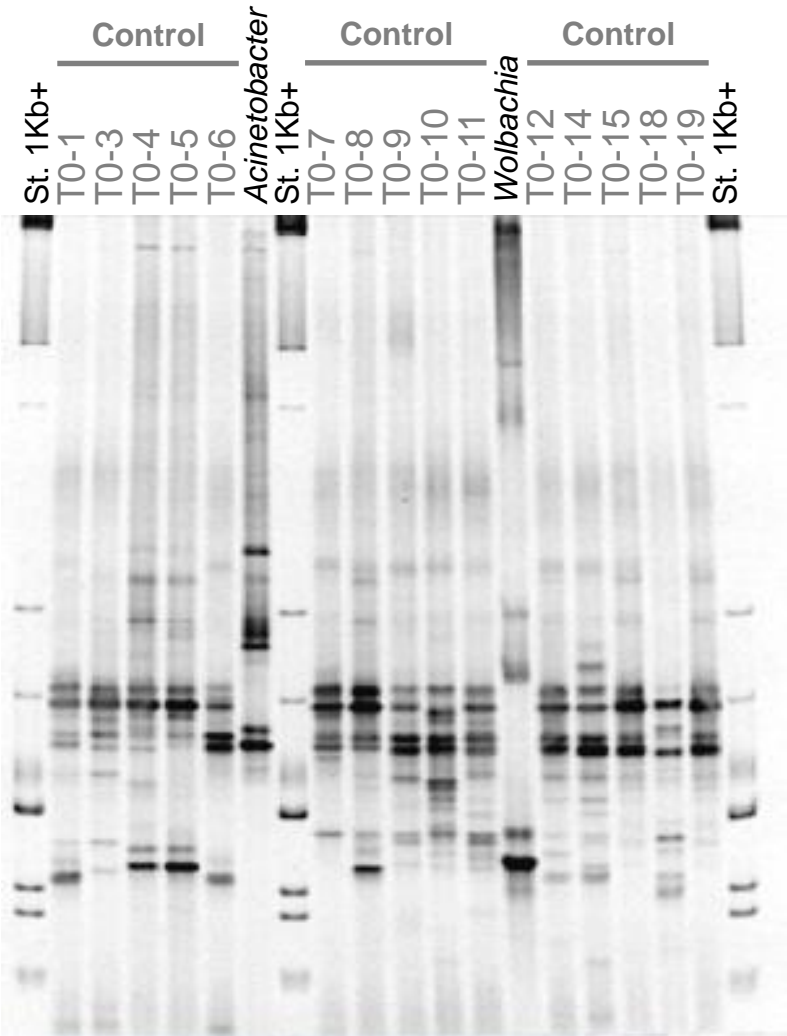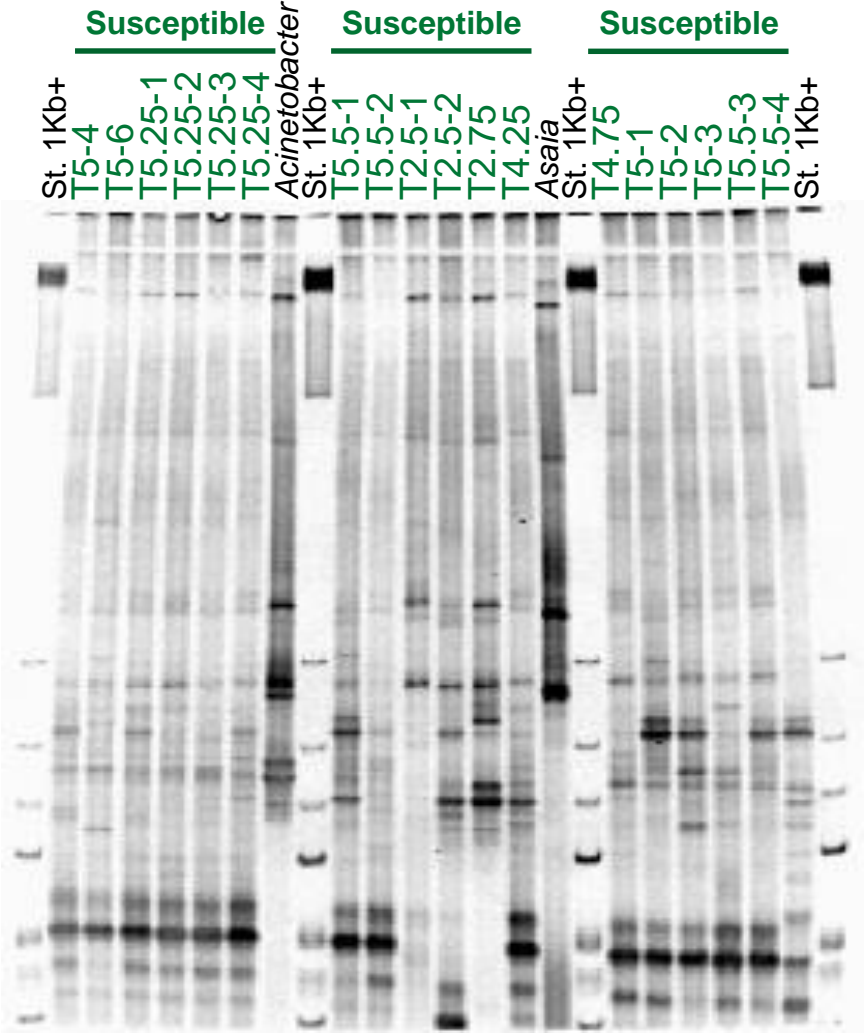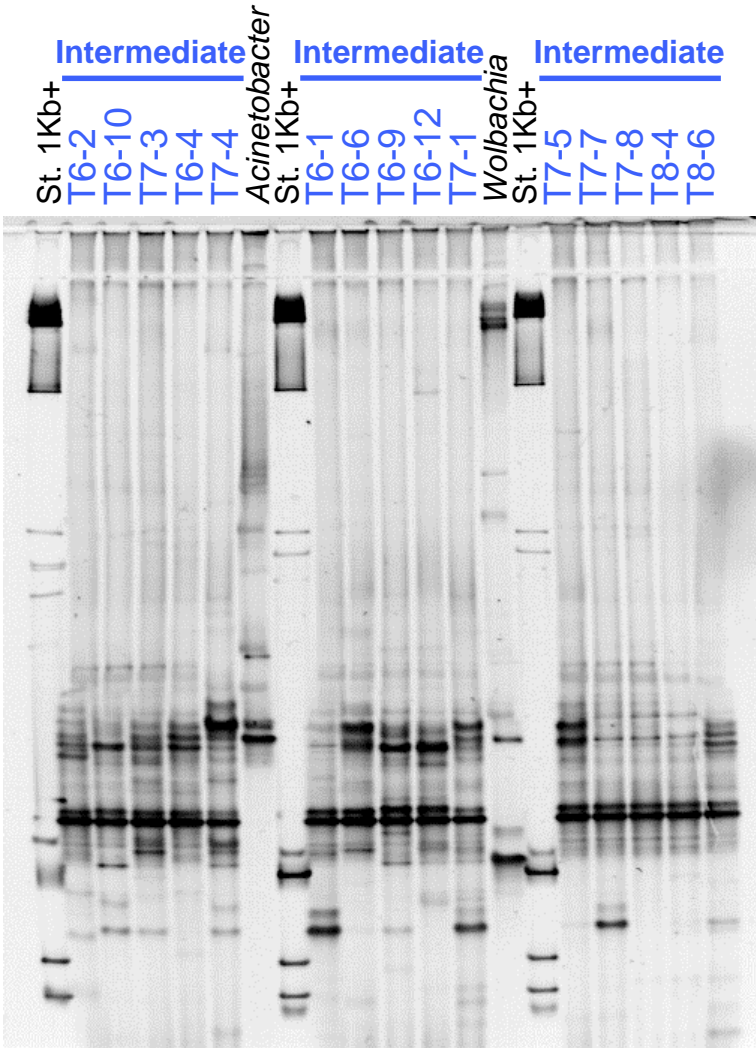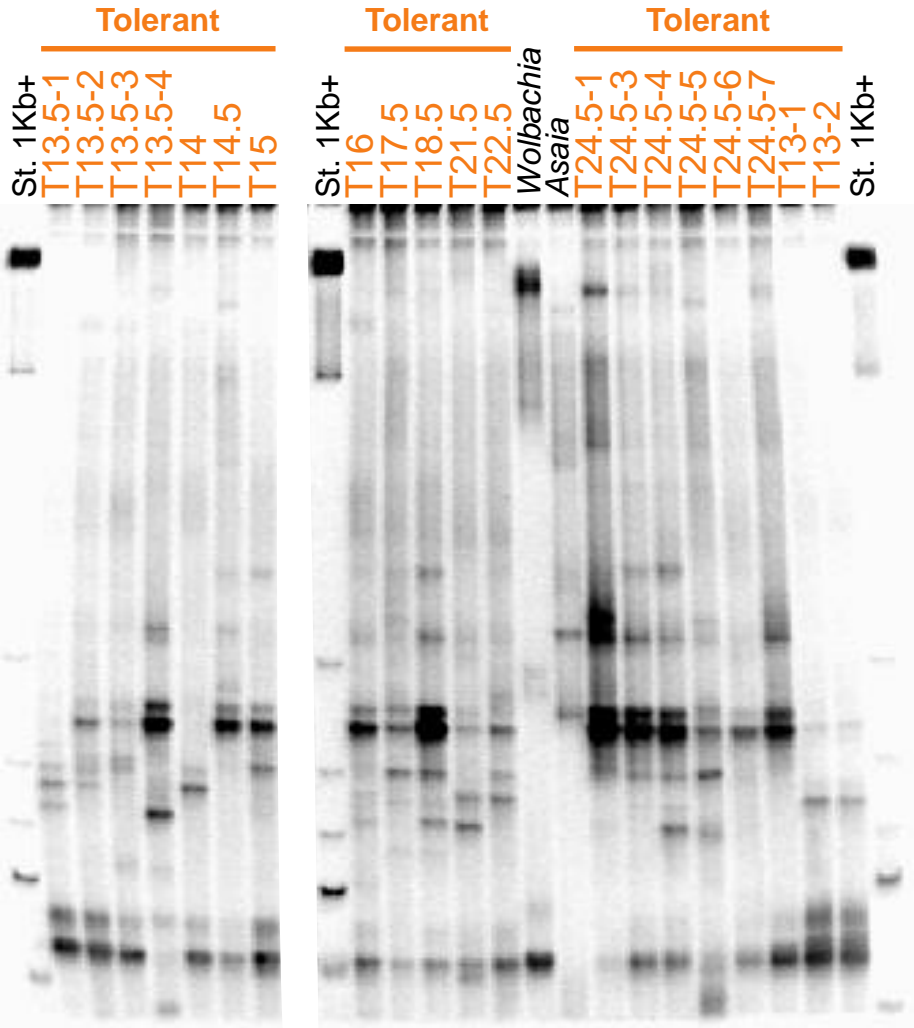

Supplement: Supplementary file 6 — Figure S3. Pictures of the four DGGE gels used in the analysis. (PDF 142 kb) [file 13071_2018_2741_MOESM6_ESM.pdf]
