# Supplementary material for: Bacterial microbiota of Aedes aegypti mosquito larvae is altered by intoxication with Bacillus thuringiensis israelensis
Source: Parasit Vectors. 2018 Mar 2;11:121. doi: 10.1186/s13071-018-2741-8 (PMC5834902; doi:10.1186/s13071-018-2741-8)
Supplement: Supplementary file 2 — Figure S2. Composite picture of a DGGE gel containing all four groups of larvae. Bands excised and sequenced are indicated by a red rectangle. The corresponding species identified are indicated in the table. (PDF 242 kb) [file 13071_2018_2741_MOESM2_ESM.pdf]

Additional file 2: Figure S2. Composite picture of a DGGE gel containing all four groups of larvae. Bands excised and sequenced are indicated by a red rectangle. The corresponding species identified are indicated in the table.

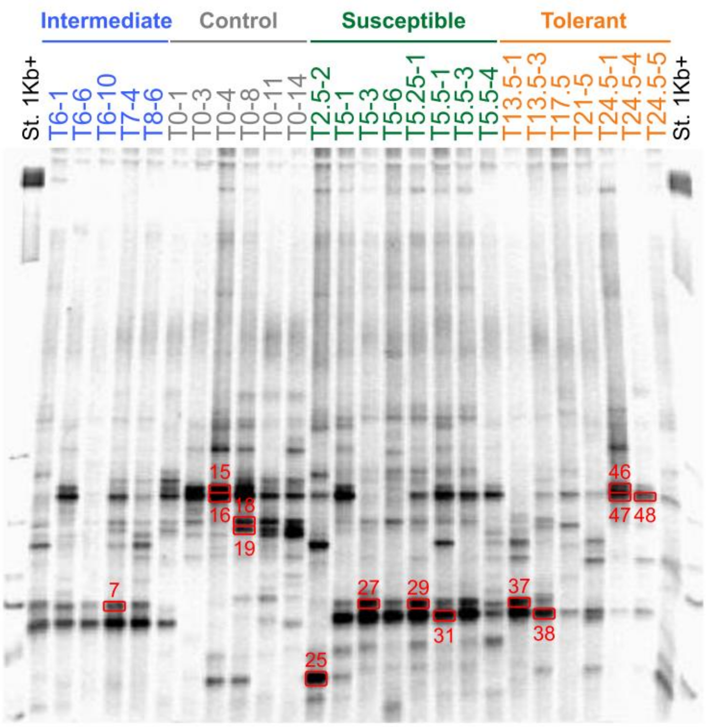

Band	Size (bp)	Phylogenetic affiliation	Most closely related organism	Accession number	Identity (%)
7	193	*Firmicutes*	*Bacillus sp.*	KC867313	99%
15	195	*Gammaproteobacteria*	*Acinetobacter sp.*	KY124158	99%
16	193	*Gammaproteobacteria*	*Acinetobacter sp.*	KM488465	97%
18	193	*Firmicutes*	*Staphylococcus sp.*	KY608158	97%
19	193	*Firmicutes*	*Staphylococcus sp.*	JQ958869	96%
25	194	*Betaproteobacteria*	*Delftia sp.*	KY419588	96%
27	195	*Firmicutes*	*Bacillus sp. (thuringiensis)*	KY608117	100%
29	196	*Firmicutes*	*Bacillus sp.*	FR821125	99%
31	195	*Firmicutes*	*Bacillus sp. (thuringiensis)*	KY608117	100%
37	195	*Firmicutes*	*Bacillus sp. (thuringiensis)*	KY608117	100%
38	195	*Firmicutes*	*Bacillus sp. (thuringiensis)*	KY608117	100%
46	195	*Gammaproteobacteria*	*Acinetobacter sp.*	KY124158	98%
47	195	*Gammaproteobacteria*	*Acinetobacter sp.*	KY124158	100%
48	195	*Gammaproteobacteria*	*Acinetobacter sp.*	KY124158	100%
