# Supplementary material for: Bacterial microbiota of Aedes aegypti mosquito larvae is altered by intoxication with Bacillus thuringiensis israelensis
Source: Parasit Vectors. 2018 Mar 2;11:121. doi: 10.1186/s13071-018-2741-8 (PMC5834902; doi:10.1186/s13071-018-2741-8)
Supplement: Supplementary file 3 — Table S1. Tukey’s post-hoc test outputs from larval bacterial microbiota. Linear models were used to test the relationship between alpha-diversity metrics (diversity) and treatment groups (treatment). Tukey’s post-hoc tests were used to compare pair of treatment (Z-tests). P-values are then reported for each model and indicated in bold when significant (P < 0.05). (PDF 195 kb) [file 13071_2018_2741_MOESM3_ESM.pdf]

Additional file 3: Table S1. Tukey's *post-hoc* test outputs from larval bacterial microbiota. Linear models were used to test the relationship between alpha-diversity metrics (diversity) and treatment groups (treatment). Tukey's *post-hoc* tests were used to compare pair of treatment (*Z*-tests). *P*-values are then reported for each model and indicated in bold when significant ($P < 0.05$).

	lme (diversity~ treatment)	control *vs* susceptible	control *vs* intermediate	control *vs* tolerant	susceptible *vs* intermediate	susceptible *vs* tolerant	intermediate *vs* tolerant
Shannon's diversity	$F_{3,63}$=49.9, **P<0.0001**	Z=4.90, **P<0.0001**	Z=4.24, **P=0.0004**	Z=5.33, **P<0.0001**	Z=0.53, P=0.95	Z=10.79, **P<0.0001**	Z=9.87, **P<0.0001**
Simpson's diversity	$F_{3,63}$=56.8, **P<0.0001**	Z=6.57, **P<0.0001**	Z=5.60, **P<0.0001**	Z=4.21, **P=0.0005**	Z=0.80, P=0.86	Z=11.41, **P<0.0001**	Z=10.19, **P<0.0001**
Pielou's eveness	$F_{3,63}$=10.0, **P<0.0001**	Z=0.61, P=0.93	Z=2.06, P=0.18	Z=3.19, **P=0.012**	Z=2.73, **P=0.040**	Z=2.65, **P=0.048**	Z=5.39, **P<0.0001**
Number of bands	$F_{3,63=}$58.6, **P<0.0001**	Z=6.31, **P<0.0001**	Z=5.28, **P<0.0001**	Z=4.77, **P<0.0001**	Z=0.90, P=0.80	Z=11.71, **P<0.0001**	Z=10.38, **P<0.0001**
