# Supplementary material for: Bacterial microbiota of Aedes aegypti mosquito larvae is altered by intoxication with Bacillus thuringiensis israelensis
Source: Parasit Vectors. 2018 Mar 2;11:121. doi: 10.1186/s13071-018-2741-8 (PMC5834902; doi:10.1186/s13071-018-2741-8)
Supplement: Supplementary file 4 — Table S2. Band counting numbers, Shannon index (diversity), Simpson index (diversity) and Pielou’s index (evenness). They are based on DGGE band-matching surfaces. Results are for larvae never exposed to Bacillus thuringiensis israelensis (Bti) (“Control”) and for larvae exposed to Bti, including larvae dead in less than 6 h of exposure to Bti (“Susceptible”), in between 6 and 11 h (“Intermediate”), and past 11 h (“Tolerant”). Results are given with mean (± standard deviation) (minimum-maximum values). (PDF 277 kb) [file 13071_2018_2741_MOESM4_ESM.pdf]

Additional file 4: Table S2. Band counting numbers, Shannon index (diversity), Simpson index (diversity) and Pielou's index (evenness). They are based on DGGE band-matching surfaces. Results are for larvae never exposed to *Bacillus thuringiensis israelensis* (Bti) ("Control") and for larvae exposed to Bti, including larvae dead in less than 6 h of exposure to Bti ("Susceptible"), in between 6 and 11 h ("Intermediate"), and past 11 h ("Tolerant"). Results are given with mean (± standard deviation) (minimum-maximum values).

	**Number of bands**	**Shannon's diversity**<br>H'= -SUM(Pi*Log(Pi))<br>Log base e	**Simpson's diversity**<br>1-λ'=1-SUM(Ni*(Ni-1)/(N*(N-1)))<br>with N = total individuals	**Pielou's evenness**<br>J'=H'/log(S)<br>with S=total species
Control	11.5 (± 2.67) (7-16)	2.4 (± 0.25) (1.9-2.8)	0.96 (± 0.02) (0.92-0.98)	1.0 (± <0.001)
Susceptible	16.6 (± 1.87) (12-19)	2.8 (± 0.12) (2.5-2.9)	0.98 (± 0.005) (0.96-0.98)	1.0 (± <0.003)
Intermediate	15.9 (± 2.42) (12-20)	2.7 (± 0.15) (2.5-3.0)	0.98 (± 0.006) (0.96-0.98)	1.0 (± <0.001)
Tolerant	7.9 (± 2.11) (4-11)	2.0 (± 0.29) (1.4-2.4)	0.93 (± 0.03) (0.84-0.96)	1.0 (± <0.005)
