# Supplementary material for: Bacterial microbiota of Aedes aegypti mosquito larvae is altered by intoxication with Bacillus thuringiensis israelensis
Source: Parasit Vectors. 2018 Mar 2;11:121. doi: 10.1186/s13071-018-2741-8 (PMC5834902; doi:10.1186/s13071-018-2741-8)
Supplement: Supplementary file 5 — Table S3. Bacterial community similarity within and between treatment groups. Similarity values are based on DGGE band-matching surface matrix. Results are presented as the mean (± standard deviation) (minimum-maximum values). (PDF 9 kb) [file 13071_2018_2741_MOESM5_ESM.pdf]

Additional file 5: Table S3. Bacterial community similarity within and between treatment groups. Similarity values are based on DGGE band-matching surface matrix. Results are presented as the mean (± standard deviation) (minimal-maximal values).

	Control	Susceptible	Intermediate	Tolerant
Control	62.0 (±11.53) (34.1-89.9)	39.2 (±9.32) (15.4-62.9)	31.1 (±7.00) (13.6-47.7)	24.0 (±11.70) (0.0-53.8)
Susceptible	/	54.2 (±11.18) (29.7-92.6)	36.4 (±8.00) (6.0-55.7)	20.4 (±9.03) (0.0-48.3)
Intermediate	/	/	69.7 (±8.14) (48.0-90.5)	34.8 (±8.58) (9.6-55.0)
Tolerant	/	/	/	40.6 (±19.33) (0.0-89.1)
