# Supplementary material for: Bacterial microbiota of Aedes aegypti mosquito larvae is altered by intoxication with Bacillus thuringiensis israelensis
Source: Parasit Vectors. 2018 Mar 2;11:121. doi: 10.1186/s13071-018-2741-8 (PMC5834902; doi:10.1186/s13071-018-2741-8)

Additional file 7: Figure S4. Non-metric multi-dimensional scaling plots of the bacterial communities of larvae based on DGGE lane analysis. 3D stress of the NMDS was 0.14. The NMDS representation is based on a Bray-Curtis dissimilarity matrix. Correspondence of sample names is indicated in the legend of Fig. 2.

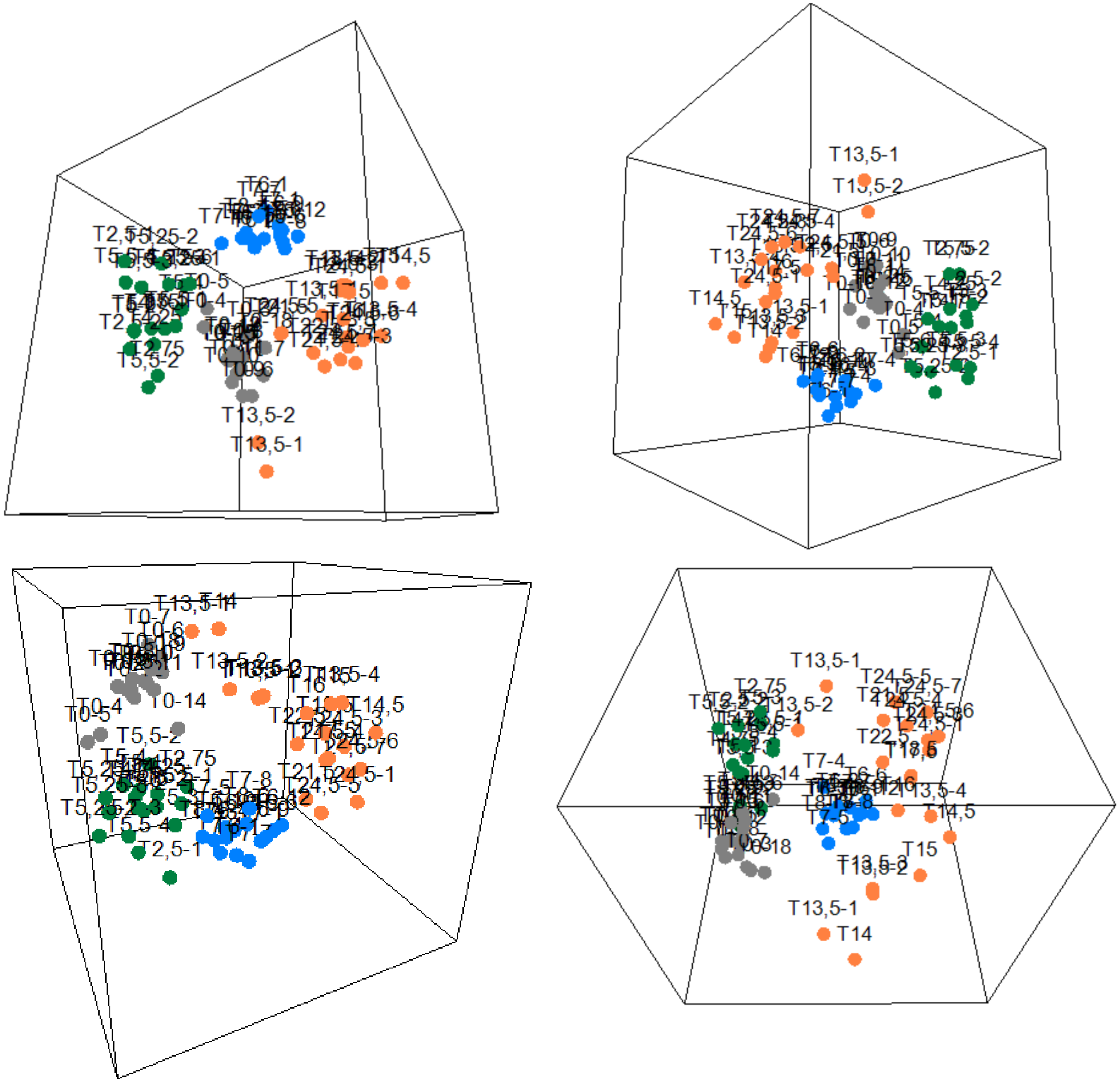

Supplement: Supplementary file 7 — Figure S4. Non-metric multi-dimensional scaling plots of the bacterial communities of larvae based on DGGE lane analysis. 3D stress of the NMDS was 0.14. The NMDS representation is based on a Bray-Curtis dissimilarity matrix. Correspondence of sample names is indicated in the legend of Fig. 2. (PDF 65 kb) [file 13071_2018_2741_MOESM7_ESM.pdf]
